# Tetrodotoxin Poisoning in Mainland France and French Overseas Territories: A Review of Published and Unpublished Cases

**DOI:** 10.3390/toxins14050351

**Published:** 2022-05-17

**Authors:** Pierrick Gouel, Clémence Mahana iti Gatti, Luc de Haro, Alice Liautaud, Jérôme Langrand, Denis Boucaud-Maitre

**Affiliations:** 1 Service des Urgences, Centre Hospitalier de Mayotte, 97600 Mamoudzou, France; pierrick.gouel@gmail.com; 2 Laboratoire des Biotoxines Marines, Institut Louis Malardé, UMR 241 EIO (Ifremer, ILM, IRD, UPF), 98713 Papeete, French Polynesia; cgatti@ilm.pf; 3 Clinical Pharmacology, Poison Control Center, St Marguerite Hospital, 13009 Marseille, France; luc.deharo@ap-hm.fr; 4 Centre Antipoison de Paris, Hôpital Fernand Widal, 75010 Paris, France; alice.liautaud@aphp.fr (A.L.); jerome.langrand@aphp.fr (J.L.); 5 Department of Research an Biostatistics, University Hospital of Guadeloupe, LAMIA (EA4540) French West Indies University, 97159 Pointe-à-Pitre, France

**Keywords:** tetrodotoxin, marine toxicology, review, France

## Abstract

Tetrodotoxin is one of the most potent neurotoxins in the aquatic world. This review of published and unpublished reports aims to describe the poisoning cases that have occurred in mainland France and overseas territories. Six articles were included, with 13 poisoning events, individuals or collective (number (*n*) = 53 patients). Moreover, 13 unpublished poisoning events from toxicovigilance networks were found (*n* = 17). All cases happened in overseas territories: French Guyana (*n* = 7), New Caledonia (*n* = 11), Reunion (*n* = 35) and French Polynesia (*n* = 17). The median age was 36 years. The most frequent signs were neurological (81.8%), digestive (54.5%) and general (52.3%). Three cases of dysgueusia and nine cases of urogenital discomfort were observed in French Polynesia. Twelve severe cases were reported, including seven deaths. Only three events (11.5%) were documented by a tetrodotoxin assay. Two families of fish accounted for 91.6% of the poisonings: 33.3% due to the Diodontidae family and 58.3% to the Tetraodontidae family. Although rare, information and collection campaigns on tetrodotoxin poisoning are, therefore, essential.

## 1. Introduction

The word ichtyosarcotoxism, from the greek *ichtyos* (fish), *sarcos* (flesh) and *toxicon* (poison) concerns the poisonings following the consumption of fresh fish. The most well-known of these poisonings is that linked to ciguatera. Scombrotoxism is the most common form of ichtyosarcotoxism worldwide, and is poisoning caused by eating the flesh of fish containing histidine. Under some bacteria’s action, histidine is decarboxylated to histamine, which can induce signs imitating allergic reactions. Many other ichtyosarcotoxisms exist, with specificities for each of them: sometimes geographic (like clupeotoxism in some seas), vectorial (like carchatoxism of the shark or the chelonitoxism of the green turtle) or syndromic (like ichthyoallyeinotoxism whose hallucinations are characteristic). Among these ichtyosarcotoxisms, tetrodotoxism has a special place because of the seriousness of the intoxications [1].

Worldwide literature reports more than 3000 patients intoxicated by tetrodotoxin (TTX) [2]. Japan is the most concerned country and is still counting more than 50 cases every year, but the mortality rate has dropped drastically [3,4]. In continental Europe, the first published case of TTX intoxication occurred in Spain in 2010 following the ingestion of a mollusc species, *Charonia saulae* [5]. Four groups of animal species are responsible for all of the worldwide poisonings, two thirds of which are secondary to the consumption of fish of the order Tetraodontiformes. The other poisonings concern gastropods, horseshoe crabs and several species of cephalopods of the genus *Hapalochlaena* [2].

TTX toxicity by ingestion has been known since the antiquity, but the mechanisms of its biosynthesis and its transport between its place of genesis and its target organs are still to be clarified. The clinical presentation depends on the age, comorbidities of the patient and the quantity of toxin ingested [4]. A TTX poisoning classification was proposed as early as 1941 by Fukuda [3,6] and remains very widely used. The first symptoms occur within 30 min after the meal. These signs are neurological and digestive. Buccal and peribuccal paresthesias appear first, followed by vertigos with nausea and vomiting. The second phase corresponds to the increase in these neuromuscular symptoms. In the third phase, cardiopulmonary signs appear, as well as the first signs of dyspnea. The fourth phase is reached when the patient presents with respiratory distress. In the “classic” clinical picture, the patient presents an ascending tetraparesis leading to asphyxia.

Treatment is symptomatic. In case of respiratory distress, mechanical ventilation support is necessary. To date, there is no antidote to TTX. The effectiveness of the anticholinesterases is controversial and does not seem to give conclusive results. [3]. When respiratory failure is palliated early enough, and the patient survives 24 h after the toxin ingestion, the prognosis is generally good [3]. The diagnosis is based primarily on anamnesis. In 87% of worldwide published cases, no TTX assay was performed [2].

France, with its overseas territories in all oceans, has often dealt with this type of poisoning but, until now, only in a very sporadic manner. No study has gathered and analyzed all the French cases reported in the literature. The aim of this study was to carry out a review of published French cases, as well as to search for unpublished cases from toxicovigilance networks: poison control centers and experts participating in this surveillance in the overseas territories (sentinel toxicologists, French Polynesia ciguatera surveillance network). The different clinical presentations and species incriminated were researched.

## 2. Results

**Figure 1:**
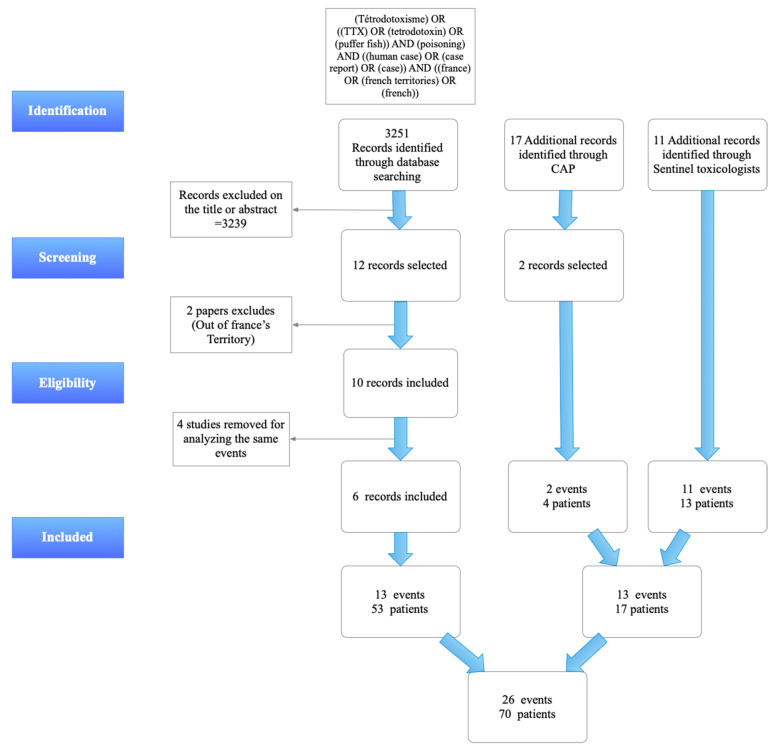
PRISMA Flow-chart of TTX poisonings in Mainland France and French Overseas Territories (CAP = Centre Antipoison or Poisoning Center).

Among the 12 articles selected on the basis of title or abstract [7,8,9,10,11,12,13,14,15,16,17,18], two articles were excluded because they corresponded to cases occurring outside French territories [14,15]. Finally, four articles mentioned intoxication events that were redundant with the other articles [7,16,17,18]. In total, six articles were included [8,9,10,11,12,13], describing 13 tetrodotoxism events involving 53 patients. The poison centers search identified 17 events. Only two events involving tetrodotoxin met the selection criteria. One concerned three patients; the other was an isolated case. The search among toxicologists in the French overseas territories revealed 11 unpublished intoxication events involving 13 patients (10 TTX poisoning events including 12 patients, reported by Louis Malardé’s Institute in French Polynesia and 1 isolated case reported by a New Caledonian toxicologist). This survey therefore allowed the identification of 13 new intoxication events, including 17 patients, which had not been published. In total, this review included 26 poisoning events, 11 of them being collective (Figure 1). The number of intoxicated persons was 70.

### 2.1. Demographic Characteristics

Of the 49 cases reporting the sex of the patients, 36 (73.5%) were male and 13 (26.5%) female. Of the 39 cases reporting age, the median age was 36 years with a minimum of 2 years and a maximum of 74 years. Five cases (10.2%) involved children (<16 years).

**Figure 2:**
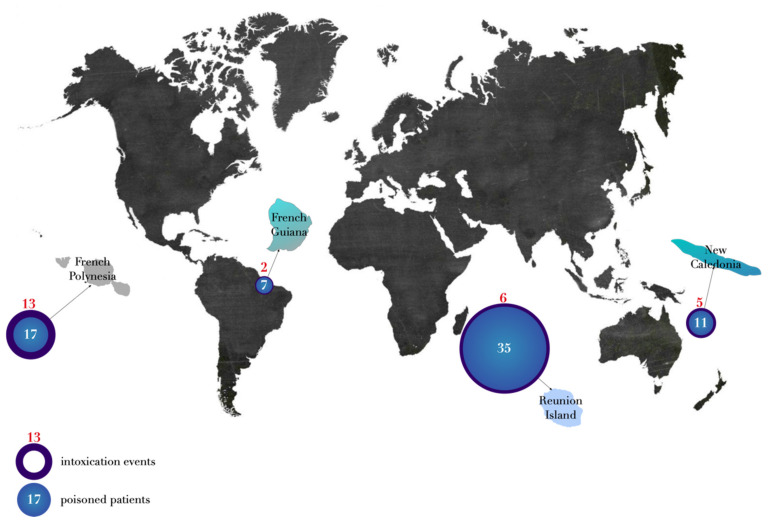
Geographic distribution of the cases.

Two events involved seven patients occurred in French Guyana; five in New Caledonia with 11 patients; six in Reunion island with 35 patients; 13 in French Polynesia including 17 patients (Figure 2). To date, no acute TTX poisoning has been reported in mainland France and French West Indies (Guadeloupe and Martinique) (Figure 2).

### 2.2. Temporal Characteristics

**Figure 3:**
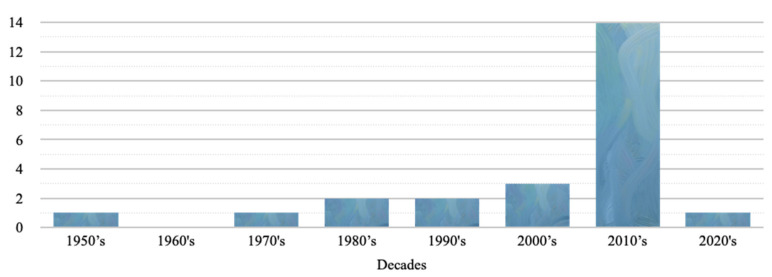
TTX poisonings reported in France by decades.

The reported poisonings ranged from the year 1959 to the year 2020. More than 75% of the reported poisonings occurred in the last two decades (Figure 3). Seventy percent of the intoxication events occurred between May and October. Of the 26 events, 21 filled out the exact date of the poisoning. 71.4% of the poisonings occurred on weekends (Friday, Saturday, Sunday), particularly on Friday (number (*n*) = 8).

### 2.3. Clinical Presentations

The mean incubation time was of 241 min (about 4 h) with a median of 180 min (3 h). The minimum was 10 min; the maximum was 16 h [8].

Clinical signs were not described for 26 patients. In total, of the 44 cases described, six (13.6%) cases were strictly asymptomatic (persons having participated in a collective poisoning meal). The clinical signs observed were neurological (*n* = 36; 81.8%), digestive (*n* = 24; 54.5%), general (*n* = 23; 52.3%), cardiological (*n* = 8; 18.2%), respiratory (*n* = 7; 15.9%), and cutaneous (*n* = 7; 15.9%). The most frequent clinical signs (more than 10% of patients) are described in Table 1. All of the clinical signs are available in Appendix A.

### 2.4. Neurologic Signs

Thirty-six patients (81.8%) presented with neurological signs. Thirty-three (75.0%) cases described paresthesias. It consisted of distal or of extremity paresthesias (*n* = 21), peribuccal (*n* = 10), labial (*n* = 4) and buccal (*n* = 3) paresthesias. Fifteen (34.1%) patients presented sensitivity disorders (including allodynia: pain experienced by usually painfree stimuli): ten Polynesian cases (“touch disorder” and “allodynia”) and five New Caledonian cases (two reported a deep sensitivity disorder and the other three, superficial sensitivity disorders). Headache was described in seven cases, ataxia in 10 cases, vestibular syndrome in two cases, mydriasis in five cases (including one with anisocoria), tetraparesis in seven cases and a loss of reflex in five cases. Two comas were observed (with a Glasgow coma scale of 3).

#### 2.4.1. Digestive Signs

Of the digestive symptoms (*n* = 24, 54.5%), nausea (*n* = 16), vomiting (*n* = 10), and diarrhea (*n* = 6) were the most frequently observed signs.

#### 2.4.2. General Signs

Sixteen (36.4%) patients reported a feeling of muscular weakness. Two cases of hypothermia were reported in the 2013 Reunion’s poisoning (33.8 °C and 35.2 °C) [9]. The other general signs described were chills (*n* = 6) and pain such as arthralgias (*n* = 4), myalgias (*n* = 2). The cases reporting a uro-genital discomfort (*n* = 3) or throat burns/dysgueusia (*n* = 9) were all from cases referred in Polynesia.

### 2.5. Cardio-Respiratory Signs

Eight (18.2%) cases reported cardiological signs including six bradycardia and seven cases with respiratory signs.

### 2.6. Cutaneous Signs

For cutaneous signs (*n* = 7, 15.9%), four of the five cases reporting itching or pruritus were from French Polynesia.

### 2.7. Severity Score

Among the 44 clinical descriptions: 12 (27.3%) patients corresponded to PSS = 3, 2 (4.5%) to PSS = 2, 24 (54.5%) to PSS = 1 and 6 (13.6%) to PSS = 0. According to the poisoning grades on the Fukuda scale [6], 12 (27.3%) cases were in the 4th stage, 11 (25.0%) in the 3rd stage, one (2.3%) case in the 2nd stage and 14 (31.8%) in the 1st stage of intoxication.

### 2.8. Treatments

Only 13 cases stated the undertaken treatment. Seven patients were intubated. Two cases required the use of catecholamines. Two patients received an activated charcoal treatment and a gastric lavage. The timing of treatment was not included in these articles. One patient received mannitol and antiemetic administration.

### 2.9. Mortality

Seven deaths (10.0%) out of the 70 patients were reported.

### 2.10. TTX Toxicological Analysis

Only three poisoning events reported a TTX toxicological assay: the 2008 French Guyanese case [11], the 2013 collective poisoning in Reunion [9], as well as the 2014 New Caledonian case [15].

### 2.11. Incriminated Animals

**Figure 4:**
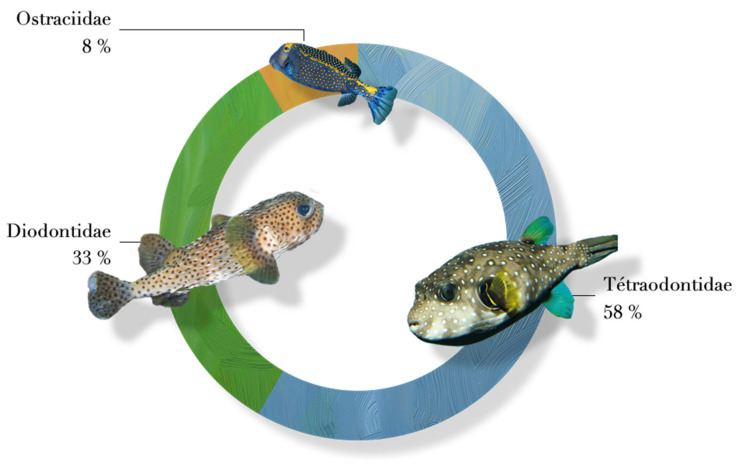
Incriminated fish families in tetrodotoxin poisoning in France.

The fish origin was only provided for five poisonings; four personal fisheries, and one secondary to purchase from a non-professional fisherman. Of the 26 poisonings, 24 events described the animal responsible for the poisoning. All of the TTX poisonings followed the intake of a fish from the Tetraodontiform order. Among this order, three families are responsible for all the poisonings (Figure 4): the Tetraodontidae (*n* = 14, 58%), the Diodondidae (*n* = 8, 33.3%) and the Ostraciidae (*n* = 2, 8%). Among the Tetraodontoidae family, four observations were found involving a fish from the Arothron genus with the *A. nigropunctatus*, *A. meleagris* and *A. hispidus* species, two observations implied a fish from the Canthigaster genus with the *C. solandri* species, three observations implied a fish from the Lagocephalus genus with the *L. sceleratus* species and finally, one observation implied a fish from the Sphoeroides genus with the *S. testudineus* species.

## 3. Discussion

Between 1959 and 2020, 70 TTX poisoning cases were reported in France. Although this poisoning is rare, an increase in the published cases has been reported since the year 2000. In the context of under-reporting of cases and a lack of systematic toxicological confirmation, it is difficult to formally conclude whether there has been an increase or decrease in cases since 2000. All of the poisoning cases happened in French overseas territories: in French Guiana, French Polynesia, New Caledonia and in Reunion island. No cases were reported in French West Indies, in Mayotte, or in mainland France. A recent review of worldwide cases [1] did not identify any cases in French Polynesia or in New Caledonia, representing 40% of the total number of cases in our series, mainly unpublished cases, and a maritime territory of 6,400,000 km^2^.

The TTX poisoning has been responsible for seven deaths (10% of our cases) and for 12 severe cases. In comparison, Japan reported a mortality rate of 8.3% in 2005 [3]. In Italy, the rate would reach 23.1% [2]. Among the clinical signs described, neurological, digestive and general signs were mainly found. In addition, some clinical signs seem to be specific to French Polynesia, notably cases of throat burn/dysgueusia or urogenital discomfort. Pruritus was also frequently found, a characteristic clinical sign of ciguatera (a poisoning associated with this territory [12]). In the absence of toxicologic analysis, it was not possible to distinguish with certainty the bio-toxin that caused these poisonings, between ciguatoxin or tetrodotoxin. A co-poisoning cannot be ruled out, although this phenomenon has been poorly documented [19].

The Diodontidae and the Tetraodontidae families were responsible for 22 of the 25 poisonings identified. Except for the 2013 case in Reunion Island [9], there was little documentation of the species involved. The species was described by the patient or by photographs presented by the health care teams. In addition, the vernacular (or generic) name could be misleading about the incriminated species. For example, the “puffer fish” is normally used to refer to the family Ostracidae. However, this vernacular name of puffer fish is cited in the title of a Moroccan publication reporting a tetrodotoxism poisoning to designate a *Lagocephalus sceleratus* (Tetraodontidae family) [14]. In our study, this “puffer fish” (Ostraciidae) has been incriminated in two TTX poisoning events. One in New Caledonia published in 1996 and the other in Reunion Island in March 2013 thanks to our Poison Centers’ investigation. The clinical presentation of this last case was more benign but required a 3-day hospital stay. This fish family is, however, considered as a non-carrier of TTX in Japan [3]. There is indeed a hemolytic toxin in the skin of this fish, called Pahutoxin, but no TTX [20]. The 1996 case presents the TTX poisoning signs with characteristic observation of a flaccid ascending tetraparesis with respiratory failure. To our knowledge, there are no other cases of tetrodotoxism implying this fish family [2]. Thus, either the fish identification was wrong; or this species could carry TTX in these regions. The Diodontidae family could also present the same ambiguity. A toxicological analysis made on a *Diodon hystrix* fished in Japan refutes the toxic nature of the fish [20,21], while other studies have shown the TTX presence in this same species [22,23].

On the background of these poisonings, the study highlights that they occur mainly on weekends (71%), particularly on Fridays (38%). This temporal characteristic could attest to a wider fish intake on this day or a festive intake at the end of the week. Indeed, ultra-mariners are, for the most part, aware of the toxicity of fish and the species incriminated. In August 2014, a New Caledonian fisherman was aware of the toxicity of fish, as was the patient in Thio in 2006 “arguing that he had previously consumed this type of fish without harm”. The same was true in 1996, when young patients admitted with “great reluctance” which fish they had consumed [11]. The last Polynesian case of 2020 was similar. The three guests claimed to have mastered the fish preparation they consumed regularly. The Hue-Hue “tender and soft” flesh has, however, killed one of the patients. It would be necessary to understand the appeal of this fish to the consuming population. Potential recreational effects of the flesh intake (known for containing only infinitesimal toxin quantities) [21,22] or the appeal of danger could be the basis of further research to consider. We observed also that 70% of cases occurred between May and October, suggesting that the toxicity could vary with the season [24].

This study has limitations. First, toxicological analysis was performed for only three events, due to the lack of leftover meals and to the lack of equipment in French overseas laboratories to assay TTX. A worldwide retrospective study suggests that 87.1% of the cases did not perform TTX detection [1]. We have selected only the cases supported by a medical history and indicating a previous consumption of seafood products linked to TTX intoxication. We cannot exclude that some cases could be due to saxitoxin, the symptoms being similar. Saxitoxin and tetrodotoxin can be isolated from the same species, including pufferfish. Regarding unpublished events (*n* = 10) occurring in French Polynesia, the field sampling campaigns leading by the marine biotoxin research laboratory (ILM) have never detected the presence of saxitoxin-producing organisms in this territory. Secondly, a search for additional cases in the overseas hospital databases could have been performed to identify potential new cases. Our series is limited to mainland France and overseas French territories. Nevertheless, the particularity of France, with overseas territories in all the tropical oceans, is both a constraint leading doctors to be trained in the management of exotic pathologies, and also an opportunity to collect data in a precise manner in areas that are very far from Europe (which is not always the case in neighboring countries where medical observations are not subject to scientific and toxicological description). Lastly, clinical signs and the amount of toxin ingested were not systematically described. Our series, including unpublished cases and potential new involved species and symptoms, may, therefore, be very useful for future global systematic review on the matter. This literature review allows us to consider public health actions. First of all, the search for TTX in species already incriminated or observed unusually in the cases mentioned in this work, would allow answering their toxicity. These analyses should, as a priority, take place on the sites where these species are consumed. Furthermore, standardized questionnaires for marine poisonings should be developed for general and emergency physicians to collect more exploitable data while providing assistance to the clinician for their diagnosis and management. Finally, an information campaign on the danger of the *Lagocephalus sceleratus* in the Mediterranean region could minimize acute TTX poisoning. TTX accumulation observed in seafood can disturb the usual clinical picture; a whole multidisciplinary work on possible shell poisonings should take place among health professionals. Even if the European Food Safety Authority (ESFA) tries to determine a minimum acceptable dose in shells, it is, to date, and given our current knowledge on the subject, impossible to set this last one: any TTX presence in seafood could present a risk for public health.

## 4. Conclusions

This review of the literature and unpublished cases highlights the lack of specificity of the clinical signs observed, the diagnosis being made, most of the time, only from the identification of the fish vector of the toxin. We have reported clinical signs specific to certain overseas French territories and regions, and we have observed that fish not known to be toxic in some parts of the world could probably be toxic in others. Information on the vectors of tetrodotoxin poisoning in France and overseas territories appears to be essential for the adequate management of this type of poisoning. To date, TTX poisonings in France have been exclusively related to the consumption of fish and have only occurred in overseas territories. Nevertheless, with the hare fish *Lagocephalus sceleratus* colonization of the Mediterranean basin, tetrodotoxin poisoning could become a real danger for Europe.

## 5. Materials and Methods

The PRISMA 2020 guideline (checklist and flowchart) was used for reporting this systematic review [25].

### 5.1. Search of Published Cases

The articles were searched using the following databases: MedLine, Scopus and Google Scholar, using the following keywords: (Tetrodotoxism) OR ((TTX) OR (tetrodotoxin) OR (puffer fish)) AND (poisoning) AND ((human case) OR (case report) OR (case)) AND ((france) OR (french territories) OR (french)).

The inclusion criteria were: 1. Study or clinical case published whatever the year of its publication until 1 January 2021; 2. Cases of ingestion occurring in mainland France and French overseas territories; 3. Cases or studies published in French or English.

Non-inclusion criteria were: venomous fish stings, other seafood poisonings for which the clinical picture, circumstances of exposure and animal species involved are different from those observed with TTX and enabled the exclusion of these cases.

### 5.2. Search of Non-Published Cases

French poison control centers, French Polynesia ciguatera surveillance network (Louis Malardé institute) and West Indies and French Guyana overseas toxicologists were investigated. Concerning the poison control centers, an extraction of the database of medical cases of the poison control centers has been carried out since its establishment in 1999, with a search by agent “Tetrodotoxin” and by animal species containing tetrodotoxin present in the national database of products and compositions.

### 5.3. Identification of the Cases

We downloaded all titles and abstracts retrieved by electronic searching to the reference management database and removed duplicates. Two review authors (PG, DBM) independently examined the remaining unique records, excluding studies that clearly did not meet the inclusion criteria and obtaining full-text copies of potentially relevant references. All cases were then reviewed by three toxicology experts (PG, DBM, JL). The imputability method [26] was applied and only cases corresponding to a “very probable”, “probable” or “possible” imputability were retained.

The following characteristics for each case were evaluated:

1. The number of intoxicated patients, mortality;
2. Location and date of intoxication;
3. The species responsible for the poisoning and, if possible, the parts consumed;
4. The symptoms, the severity according to the Poisoning Severity Score (PSS) [27] and the evolution;
5. Tetrodotoxin toxicological analysis.

### 5.4. Statistics Analysis

Quantitative variables were expressed as median or mean ± standard deviation (SD) and qualitative variables as number and percentage.

### 5.5. Ethics

Institutional Review Board Statement: This study has been carried out in accordance with state laws regulating research involving human participants and with the Helsinki declaration. The study was approved by the Guadeloupe Ethics Committee on 1 July 2021 (Reference. A63_21_09_13_TETRODOTOXINE).

## Figures and Tables

**Table 1:** Most frequent clinical signs and symptoms (more than 10% of the patients) of the TTX poisonings in Mainland France and French Overseas Territories (*n* = 44).

Signs and Symptoms		Number of Cases (*n* = 44) and Percentage (%)
**Total neurologic signs**		**36 (81.8%)**
	• Paresthesia	33 (75.0%)
	• Sensitivity disorder	15 (34.1%)
	• Vertigo	11 (25.0%)
	• Ataxia	10 (22.7%)
	• Dysarthria	8 (18.2%)
	• Headache	7 (15.9%)
	• Tetraparesis	7 (15.9%)
	• Reflex disorder	6 (13.6%)
	• Mydriasis	5 (11.4%)
**Total gastrointestinal signs**		24 (54.5%)
	• Nausea	16 (36.4%)
	• Vomiting	10 (22.7%)
	• Abdominal pain	7 (15.9%)
**Total general signs**		23 (52.3%)
	• Muscle weakness	16 (36.4%)
	• Arthralgias, myalgias	6 (13.6%)
	• Shivering	6 (13.6%)
	• Others	9 (20.4%)
**Total cardiologic signs**		8 (18.2%)
	• Bradycardia	6 (13.6%)
**Total respiratory signs**		7 (15.9%)
**Total cutaneous signs**		7 (15.9%)
	• Pruritus	5 (11.4%)

## Data Availability

The data regarding published cases presented in this study are openly available in pubmed, reference number [8,9,10,11,12,13]. The data presented in this study for unpublished cases from Poisoning Center are available on request from the corresponding author.

## Appendix A

**Table A1:** List of clinical signs and symptoms of the TTX poisonings in Mainland France and French Overseas Territories (*n* = 38 symptomatic patients).

		Number of Cases	Percentage/Category	Percentage/Total
**Total neurologic signs**		**36**		**94.7%**
Paresthesia		**33**	91.7%	86.8%
	Distal or of the extremities paresthesias	21	58.3%	55.3%
	Peribuccal paresthesia	10	27.8%	26.3%
	Labial paresthesia	4	11.1%	10.5%
	Buccal paresthesia	3	8.3%	7.9%
	Dermatologic paresthesia	3	8.3%	7.9%
Sensitivity disorder		15	41.7%	39.5%
	“Touch disorder”	9	25.0%	23.7%
	Allodynia	6	16.7%	15.8%
	Superficial sensitivity disorder	3	8.3%	7.9%
	Deep sensitivity disorder	2	5.6%	5.3%
Ataxia		10	27.8%	26.3%
	Walking disorder	4	11.1%	10.5%
	Coordination disorder	3	8.3%	7.9%
	Equilibrium disorder	2	5.6%	5.3%
Reflex disorders		6	16.7%	15.8%
	Areflexia	5	13.9%	13.2%
	Corneal reflex abolition	1	2.8%	2.6%
Mydriasis		5	13.9%	13.2%
	Anisocoria	1	2.8%	2.6%
Vertigo		11	30.6%	28.9%
Dysarthria		8	22.2%	21.1%
Tétraparésie		7	19.4%	18.4%
Headache		7	19.4%	18.4%
Vision disorder		3	8.3%	7.9%
Nystagmus		2	5.6%	5.3%
Vestibular syndrom		2	5.6%	5.3%
Coma		2	5.6%	5.3%
**Total digestive signs**		**24**		**63.2%**
Nausea		16	66.7%	42.1%
Vomiting		10	41.7%	26.3%
Diarrhea		6	25.0%	15.8%
Hypersialorrhea		3	12.5%	7.9%
Pain		7	29.2%	18.4%
	Epigastralgic	4	16.7%	10.5%
	Abdominal	3	12.5%	7.9%
**Total general signs**		**23**		**60.5%**
Muscular weakness		16	69.6%	42.1%
Pain		6	26.1%	15.8%
	Arthralgias	4	17.4%	10.5%
	Myalgias	2	8.7%	5.3%
Shiverings		6	26.1%	15.8%
Faintness		4	17.4%	10.5%
Hypothermia		2	8.7%	5.3%
Drowsiness		2	8.7%	5.3%
Anxiety		1	4.3%	2.6%
Others		9	39.1%	23.7%
	Uro-genital discomfort	3	13.0%	7.9%
	Buccal/throat burns/dysgueusia	8	34.8%	21.1%
**Total cardiologic signs**		**8**		**21.1%**
Bradycardia		6	75.0%	15.8%
Hypotension		4	50.0%	10.5%
Tachycardia		1	12.5%	2.6%
**Total respiratory signs**		**7**		**18.4%**
Dyspnea		4	57.1%	10.5%
Respiratory distress		2	28.6%	5.3%
Desaturation		2	28.6%	5.3%
Bradypnea		2	28.6%	5.3%
**Total dermatologic signs**		**7**		**18.4%**
Pruritus		5	71.4%	13.2%
Sweating/excessive sweating		3	42.9%	7.9%
